# Supplementary material for: Polyketide synthase gene clusters in the ericoid mycorrhizal fungus Oidiodendron maius: genome-wide prediction, expression profiling, and functional characterization of knockout mutants
Source: Front Plant Sci. 2026 Sep 16;17:1924430. doi: 10.3389/fpls.2026.1924430 (PMC13623903; doi:10.3389/fpls.2026.1924430)
Supplement: Supplementary file 1 [file SupplementaryFile1.zip › Supplementary Materials/Supplementary Folder 1.zip!/Oidma1_AssemblyScaffolds_Repeatmasked/knownclusterblast/cluster23/jgi.p_Oidma1_42767_mibig_hits.html]

| MIBiG Protein | Description | MIBiG Cluster | MiBiG Product | % ID | % Coverage | BLAST Score | E-value |
| --- | --- | --- | --- | --- | --- | --- | --- |
| BAV32147.1 | geranylgeranyl\_diphosphate\_synthase | BGC0001373 | Sordarin\_biosynthetic\_gene\_cluster | 37 | 60.7619047619 | 212 | 1.7e-54 |
| AAK11531.1 | PaxG | BGC0001082 | Paxilline\_biosynthetic\_gene\_cluster | 37 | 58.2857142857 | 206 | 9.2e-53 |
| AAT65717.1 | geranylgeranyl\_diphosphate\_synthase | BGC0000629 | Aflatrem\_biosynthetic\_gene\_cluster | 38 | 57.7142857143 | 195 | 2.1e-49 |
| AAW88510.1 | geranylgeranyl\_diphosphate\_synthase | BGC0001081 | Lolitrem\_biosynthetic\_gene\_cluster | 37 | 56.9523809524 | 193 | 6.2e-49 |
| ctg1\_orf003 |  | BGC0000688 | Copalyl\_diphosphate\_biosynthetic\_gene\_cluster | 38 | 53.7142857143 | 192 | 1.1e-48 |
| CBF85177.1 | conserved\_hypothetical\_protein | BGC0000673 | Ent-pimara-8(14),15-diene\_biosynthetic\_gene\_cluster | 33 | 59.2380952381 | 185 | 1.7e-46 |
| CAA75568.1 | geranylgeranyl\_diphosphate\_synthase | BGC0001270\_c3 | Gibberellin\_A3\_/\_gibberellin\_A1\_/\_gibberellin\_A7\_/\_gibberellin\_A4\_/ | 42 | 41.7142857143 | 183 | 4.9e-46 |
| BAD29970.1 | geranylgeranyldiphosphate\_synthase | BGC0000676 | Aphidicolin\_biosynthetic\_gene\_cluster | 35 | 55.4285714286 | 175 | 1.7e-43 |
| CAP07654.1 | geranylgeranyldiphosphate\_synthase | BGC0001269\_c2 | Gibberellin\_A3\_/\_gibberellin\_A1\_/\_gibberellin\_A7\_/\_gibberellin\_A4\_/ | 34 | 56.1904761905 | 172 | 1.1e-42 |
| BAM84046.1 | prenyl\_transferase | BGC0001260 | Terpendole\_E\_biosynthetic\_gene\_cluster | 27 | 44.9523809524 | 98 | 2.7e-20 |
| AAT65718.1 | prenyl\_transferase | BGC0000629 | Aflatrem\_biosynthetic\_gene\_cluster | 26 | 43.2380952381 | 80 | 5.8e-15 |
| ACI04497.1 | CrtE | BGC0000646 | Carotenoid\_biosynthetic\_gene\_cluster | 26 | 37.9047619048 | 57 | 6.9e-08 |
